# Chemotherapy and Immunosuppressant Therapy-Induced Posterior Reversible Encephalopathy Syndrome

**DOI:** 10.7759/cureus.11163

**Published:** 2020-10-25

**Authors:** Gurleen Kaur, Ibtisam Ashraf, Mercedes Maria Peck, Ruchira Maram, Alaa Mohamed, Diego Ochoa Crespo, Bilal Haider Malik

**Affiliations:** 1 Neurology, California Institute of Behavioral Neurosciences & Psychology, Fairfield, USA; 2 Internal Medicine, Shalamar Institute of Health Sciences, Lahore, PAK; 3 Internal Medicine, California Institute of Behavioral Neurosciences & Psychology, Fairfield, USA; 4 Internal Medicine, Arogyasri Healthcare Trust, Hyderabad, IND; 5 Internal Medicine, Memorial Hermann Texas Medical Center, Houston, USA; 6 Internal Medicine, Clinica San Martin, Azogues, ECU

**Keywords:** posterior reversible encephalopathy syndrome (pres), chemotherapy, tacrolimus, immunosuppressant, radiological findings in pres

## Abstract

Posterior reversible encephalopathy syndrome (PRES) is an entity which is characterized by acute to subacute onset of neurological symptoms like altered mental status, seizures, headaches and other focal neurological deficits. It is diagnosed with the help of MRI findings which typically involve the subcortical white matter of parieto-occipital lobes. In this review, we will discuss the various etiologies and risk factors including some of the most common chemotherapeutic agents and immunosuppressant agents associated with this disorder. We will discuss the mechanism of actions and side effect profiles of a few drugs and their role in causation of PRES. This review article discusses if there is any difference in presentation and imaging findings of PRES caused by cytotoxic agents versus caused by other etiologies. It also highlights the difficulty in management of PRES caused by cytotoxic agents as the discontinuation of these drugs could be life-threatening due to graft rejections or graft versus host disease.

## Introduction and background

Posterior reversible encephalopathy syndrome (PRES), first described by Hinchey et al. in 1996, is a neurological disorder characterized by a constellation of clinical and radiological findings. There is a wide spectrum of clinical presentations ranging from transient focal neurological signs, altered consciousness, seizures to visual disturbances and headaches but can vary from case to case [1]. It is most commonly associated with other disorders like hypertension, renal diseases, autoimmune disorders, pre-eclampsia/eclampsia and cytotoxic substances. Several cancer chemotherapy drugs and immunosuppressant drugs have been reported to be responsible for PRES. Cyclosporine, tacrolimus, paclitaxel, oxaliplatin, cisplatin, gemcitabine, bevacizumab, sunitinib, and sorafenib are among a few of the most commonly associated drugs [1,2].

Eight percent of patients receiving tacrolimus immunosuppression after solid organ transplant are at risk of developing severe neurotoxicity. The cumulative incidence of tacrolimus associated-PRES in patients who underwent hematopoietic stem cell transplant was found to be 1.6%. The patients on tacrolimus or bevacizumab therapy also have a high propensity of developing hypertension as an adverse effect of the medication [3]; thus hypertension and direct cytotoxicity by the drug, both play a role in causing PRES. So, the pathophysiologic mechanisms behind this syndrome are not straightforward and more than one etiologic factor may be responsible for the precipitation of PRES.

The diagnosis of PRES is made by correlating the clinical signs and symptoms with radiological findings. Although the MRI may also show frontal lobes or basal ganglia involvement, PRES findings are typically described as vasogenic edema of bilateral subcortical white matter of the posterior hemispheres (parietal and occipital lobes) [4]. Hypertension is thought to be the primary culprit of precipitating PRES. It is thought to be caused by the failure of autoregulatory blood pressure control mechanisms due to severe hypertension leading to hyperperfusion; hence resulting in endothelial disruption causing vasogenic edema. But interestingly 15-20% of the cases were noted to have normal or below normal blood pressure readings [5]. Angiogenesis inhibiting drugs, calcineurin inhibitors, taxanes, antimetabolites, antineoplastic drugs, and toxic substances like cocaine are thought to have complex mechanisms of precipitating PRES causing endothelial damage resulting in edema which might or might not be dose-dependent. In most of the cases, PRES resolves without any serious complications if treated promptly by managing the blood pressure, immediately identifying and removing the offending agent, by treating the underlying condition and symptomatic treatment like antiepileptics [6].

The scope of this article is to learn about drug induced PRES, various etiologic factors, typical and atypical clinical presentations as well as imaging findings, and management of the disorder by reviewing the literature from case reports, case series, review articles, and retrospective studies.

## Review

Drugs causing PRES and mechanism of action

Tacrolimus and cyclosporine are calcineurin inhibitors indicated usually after solid organ and hematopoietic stem cell transplants. These agents bind with specific immunophilins (FK506 for tacrolimus) and inhibit the calcineurin dependant pathway, which in turn inhibits the proliferation of T cells. Renal impairment, cardiovascular events, abnormalities in glucose metabolism, new-onset hypertension, neurologic disorders are some of the commonly occurring adverse effects of these drugs. The neurologic side effects generally include tremors, insomnia and paraesthesia [7]. Studies have shown that it does not take supratherapeutic levels of tacrolimus to cause PRES and average levels were found to be below 15 ng/dl [3]. Tacrolimus being lipophilic does not usually cross the blood-brain barrier because it gets pumped out by P-glycoprotein efflux pumps. But due to polymorphisms in multidrug resistance genes, there might be failure or reduction in the function of these pumps causing tacrolimus to cross the blood-brain barrier [8]. Another theory states that, in transplant patients due to graft versus host reactions, inflammatory mediators like interleukin-1 and tumor necrosis factor α (TNFα) cause systemic vasoconstriction adding to the effect of tacrolimus in precipitating PRES [9]. Also, there is 35% rise in mean arterial pressure after tacrolimus regimen [1].

Gemcitabine is a nucleoside analog and acts by inhibiting DNA synthesis. It has uncommon neurological side effects like peripheral neuropathy (3%) and somnolence (9%). The first reported case of PRES with gemcitabine was in 2001 [10]. It has a propensity to induce thrombotic microangiopathy which might be caused due to disruption of the complement pathway. Gemcitabine-induced PRES is thought to be related to microvascular injury or secondary to thrombotic microangiopathy [11].

Vascular endothelial growth factor (VEGF) has a key role in angiogenesis and helps in the division and metastasis of the cancer cells. VEGF inhibitors like bevacizumab, a humanized monoclonal antibody, hinders the interaction of VEGF and VEGF receptor by binding to VEGF-A, which is a subtype of VEGF. The most common side effect of this drug is hypertension (16-38%) and is dose-dependent [12]. Other side effects are proteinuria, bleeding, thrombosis, delayed wound healing and gastrointestinal perforation. The most common use of this drug is in metastatic colon cancer, lung, breast, and ovarian cancer [13]. Drugs like sorafenib and sunitinib are tyrosine kinase inhibitors that act by inhibiting VEGF. Similar to bevacizumab, these drugs also have been reported in association with PRES.

Oxaliplatin alone and in combination with 5-fluorouracil and bevacizumab have been reported to cause PRES [14]. Oxaliplatin and cisplatin are anti-neoplastic drugs acting by inhibition of DNA replication.

In a study of 44 patients on the EPOCH (etoposide, prednisone, vincristine, cyclophosphamide and doxorubicin) regimen, three patients developed PRES [15].

Table 1 shows the list of some common drugs that have been associated with the use of PRES.

**Table 1:** Drugs associated with posterior reversible encephalopathy syndrome (PRES)

Chemotherapeutic drugs	Immunosuppressant drugs
Bevacizumab	Tacrolimus
Sunitinib	Sirolimus
Sorafenib	Cyclosporine
Cisplatin	Methotrexate
Oxaliplatin	Rituximab
Cytarabine	Intravenous Immunoglobulin
Gemcitabine	Cyclophosphamide
Vincristine	Interferon alpha
Docetaxel	Ipilimumab
Paclitaxel	
Irinotecan	

Pathophysiology

**Figure 1:**
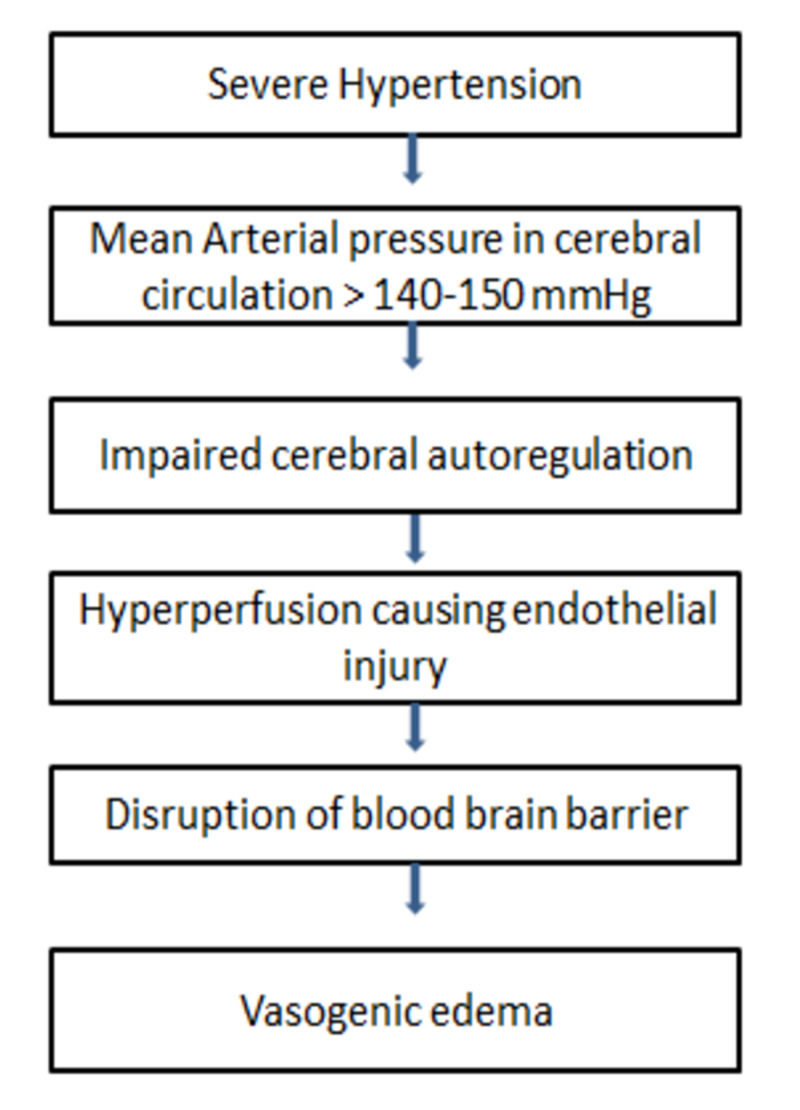
Pathophysiology of vasogenic edema in posterior reversible encephalopathy syndrome (PRES)

Several theories have been implicated in causing PRES:

1. Vasogenic theory: This theory bases on the role of severe hypertension and failure of autoregulatory mechanisms (Figure 1). It is one of the most accepted theories but this theory is put to question as the retrospective studies have shown 50% of the patients with PRES did not have severe hypertension prior to presentation [16].

2. Cytotoxic theory: It is believed that exogenous toxins and chemicals might cause direct toxicity to the endothelium of the cerebral blood vessels resulting in the disruption of the blood-brain barrier. The chemotherapeutics like vincristine and others as mentioned above, and immunosuppressant drugs like tacrolimus probably precipitate PRES via this mechanism [16].

3. Immunogenic theory: Immunogenic theory says that due to T cell activation and cytokine or vasoactive substances like histamine and nitric oxide release may cause endotheliopathy. This phenomenon might be responsible for PRES in patients with sepsis and autoimmune disorders [1,16].

4. Neuropeptide theory: Severe cerebral vasoconstriction caused by the release of mediators like endothelin 1 and thromboxane A2 might cause endothelial dysfunction and resulting edema [1,16]. 

Clinical presentation

It presents as acute onset of neurological symptoms. Seizures and headache are the most common symptoms. Other common symptoms include visual disturbances, altered mental status, hemiparesis, aphasia, ataxia and other focal deficits. A retrospective study done on 151 patients with PRES, symptom frequency was noted to be seizures in 66% followed by visual disturbances seen in 30% patients, altered mental status in 40% and paresis in 12% patients [17].

Mostly, with prompt and adequate treatment this disorder is fully reversible with very few cases with poor outcome. PRES mortality rates have been found to be somewhere between 8% and 19% [18]. In a study of 31 cancer patients (on chemotherapy/immunosuppressants) with the diagnosis of PRES, frequency of the symptoms was noted to be altered mental status in 71% patients, seizures in 58%, headache in 48%, visual disturbances in 26%. Median systolic blood pressure (SBP) and diastolic blood pressure (DBP) on the day of symptom onset were recorded to be 190 mmHg and 100 mmHg, respectively. Seventeen patients were receiving chemotherapy within one month of PRES onset. Forty-two percent were on a cytotoxic agent therapy (docetaxel, carboplatin, oxaliplatin, vincristine, paclitaxel, irinotecan), 23% were receiving a biologic agent (bevacizumab) and 38% were on a combination regimen. Four patients who underwent stem cell transplant were receiving immunosuppression with tacrolimus, cyclosporine or everolimus [19].

Most of the patients with PRES on tacrolimus therapy show a normal therapeutic range of serum tacrolimus. In a study of 19 patients on tacrolimus for stem cell transplant, it was seen that only 21.1% of the patients showed supratherapeutic levels of the drug [9]. In another retrospective study of 10 patients, cerebrospinal fluid (CSF) levels of only one patient was available and it was found to be 42 ng/ml; much higher than the corresponding serum tacrolimus levels [3]. Enough data is not available to suggest this fact but it is thought that higher levels might be due to the concentration of the drug in the brain due to the disruption of the blood-brain barrier. Tacrolimus not only predisposes to hypertension but also causes nephrotoxicity. Renal dysfunction might be an independent risk factor in PRES development. In the above mentioned study of 19 patients, serum creatinine and weight were noted to have increased an average of 62.8% and 6.8%, respectively, between baseline measurements and PRES onset [9]. One of the case review literature states that 10% rise in weight from baseline and creatinine rise of >0.16 mmol/L are a significant risk factor for PRES onset [20]. Another study of 15 patients with systemic lupus erythematosus (SLE) and on immunosuppressive medications, creatinine of >2 mg/dL was found to be the most significant risk factor for the development of PRES [21].

Visual disturbances can range from decreased vision and loss of vision to visual hallucinations. In a case reported, an SLE patient developed PRES and presented with headache, papilledema and hemorrhages in both eyes after first dose of rituximab [22].

Diagnosis and radiological findings

Posterior reversible encephalopathy syndrome is a diagnosis of exclusion and is based on clinical judgement. Clinical presentation with MRI findings in the setting of hypertension, renal dysfunction and on-going/prior chemotherapy or immunosuppression, raise the suspicion of PRES. Furthermore, various underlying risk factors and associated etiologies should be kept in consideration when PRES is suspected (like autoimmune disorders, cancer, eclampsia/pre-eclampsia). Fugate et al. proposed a diagnostic criteria which says that the presence of more than one acute neurological symptom (including seizure, encephalopathy or confusion, headache, visual disturbances) with addition to the presence of more than one risk factor (severe hypertension or blood pressure fluctuations, renal failure, immunosuppressant therapy or chemotherapy, eclampsia, autoimmune disorder) with MRI findings suggestive of PRES warrants the diagnosis of PRES provided there is no alternate diagnosis that fits into the situation [23].

MRI is the most sensitive tool in the diagnosis. During continuous electroencephalogram (EEG) monitoring, 62% patients (n=32) were found to have nonconvulsive seizures and epileptiform discharges predominantly in the posterior region [24]. In patients on chemotherapy or immunosuppression, lumbar puncture can be of importance to rule out brain metastasis or infections. In a study of 87 patients, elevated CSF levels of albumin and an elevated CSF to serum albumin ratio has been reported suggesting disruption of blood-brain barrier [25].

Typical radiologic findings include T2-FLAIR (fluid attenuated inversion recovery imaging) hyperintensities in the parietal and occipital lobes due to vasogenic edema. The nature of lesions of PRES is usually independent of underlying etiology. The Berlin study of 154 patients, reported that there is no association between the extent of edema as well as the lesion distribution to the type of toxic association with PRES [26]. This is in-line with another study of 28 patients which suggested that there is no remarkable difference in PRES caused due to different causes [27]. This suggests that there is no specific difference in radiologically diagnosed PRES due to chemotherapy/immunosuppression versus other causes. There was a significant difference reported in the mean arterial pressure (MAP) among different associations. A lower MAP was seen in chemotherapy and immunosuppression associated PRES while a higher MAP was seen with other etiologies like autoimmune disorders and infections [26].

**Figure 2:**
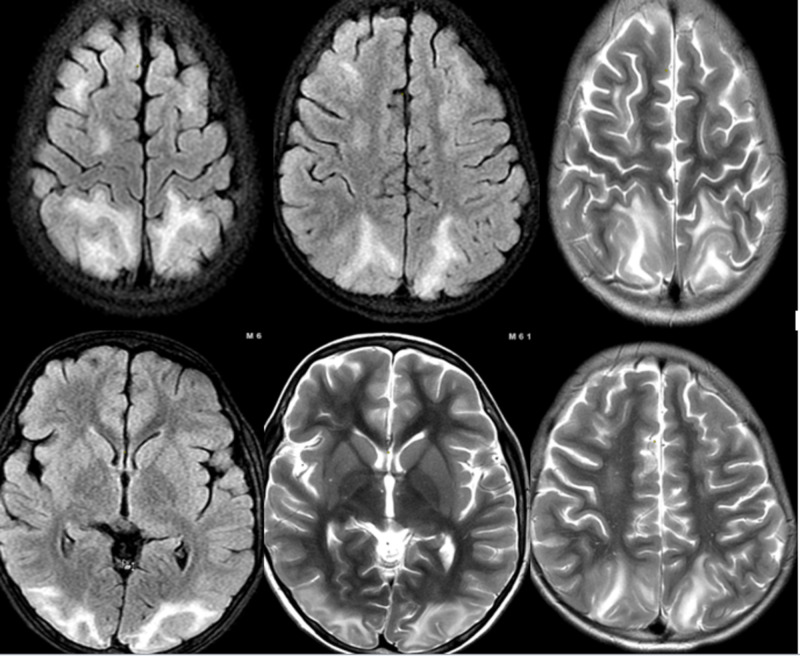
T2 FLAIR (fluid attenuated inversion recovery imaging) image showing the involvement of subcortical and deep white matter of bilateral parieto-occipital lobes, right temporal lobe and bilateral frontal lobe

Involvement of occipital lobes is the most commonly seen (≥98%) but frontal lobe involvement is not uncommon (≈70%), temporal lobe (≈65%) and cerebellum in 30 to 53% of times [28,29]. Predominantly affecting the subcortical and cortical white matter, PRES might also affect the deep white matter along with the above two in 18 to 29% of cases [27,28-29]. Three patterns of involvement have been described including superior frontal sulcus, holohemispheric watershed and dominant parieto-occipital patterns [27,28-30]. Involvement of thalamus, basal ganglia and brainstem has also been noted [30]. Figure 2 shows the involvement of subcortical and deep white matter of bilateral parieto-occipital lobes, right temporal lobe and bilateral frontal lobe. On MRI, findings suggestive of minute hemorrhages, subarachnoid haemorrhage or rarely intraparenchymal hematomas might be identified [31].

Management

Treatment of PRES is primarily symptomatic. Control of blood pressure levels by 25% is recommended [1]. Anticonvulsant treatment might be required but is usually tapered off when the patient becomes asymptomatic and MRI findings resolve [32]. In case of immunosuppression induced or chemotherapy-induced PRES, offending agent must be eliminated if possible or dose reduction can be considered if immediate discontinuation is not possible. In a retrospective study, there was no significant difference in the deaths in three treatment groups, i.e. patients with continued tacrolimus therapy (25%), holding the therapy (30%), switching to a different therapy (25%) [9]. Switching of the therapy might precipitate new onset graft versus host disease in transplant patients. So, risk to benefit ratio must be weighed by the physicians. In a retrospective study of 31 patients with cancer, successful resuming of the chemotherapy was noted without recurrence of PRES and it was suggested that chemotherapy could be safely re-administered provided careful clinical monitoring is done [12,19].

For future studies

The major limitation to this review was the lack of any prospective studies for this entity. Most of the data is based on retrospective cohort studies, case reviews and case reports. There is a need to do further research on CSF studies, diagnostic/prognostic biomarkers or definitive imaging findings to draw better guidelines for the diagnosis of this disorder. Another thing which we would like to point out at is to have further studies on comparison of CSF levels of the drugs with corresponding serum levels after PRES occurs. Higher CSF levels of the drugs that cross blood-brain barrier minimally could suggest the disruption of blood-brain barrier and help in the diagnosis of PRES.

## Conclusions

PRES is a clinicoradiological entity seen in association with hypertension, renal failure, pre-eclampsia, autoimmune disorders, chemotherapy, and immunosuppressant drugs. Sometimes the exact underlying etiology remains unclear. Due to autoregulatory failures and disruption of the blood-brain barrier, cerebral vasogenic edema occurs. The patients with drug-induced PRES are generally patients with cancer, organ transplant/stem cell transplant, or patients with autoimmune disorders. The patients usually have a therapeutic range of drug concentrations which implies dose-independent precipitation of PRES. Careful exclusion of differential diagnoses should be done by ruling out metastasis, infections, or strokes due to hypercoagulable states. Diagnosis is based on clinical and radiological findings. Management is mostly symptomatic and careful monitoring of the dose administration/discontinuation of the offending agent. There is room for further research to have a better concept of the pathophysiology, better guidelines for diagnosis, and treatment of this disorder.
